# Association of blood manganese levels with non-alcoholic fatty liver disease in NHANES 2017–2020: A retrospective cross-sectional study

**DOI:** 10.1016/j.metop.2025.100358

**Published:** 2025-03-21

**Authors:** Qian Xue, Hongju Chen

**Affiliations:** a Department of Hepatobiliary Surgery, People's Hospital of Leshan, Sichuan, China; b Department of Gynecology, Leshan Hospital of Traditional Chinese Medicine, Sichuan, China

**Keywords:** Blood manganese, NHANES, NAFLD

## Abstract

**Objective:**

This study investigates the link between blood manganese (Mn) levels and non-alcoholic fatty liver disease (NAFLD) in a U.S. adult population.

**Background:**

The role of manganese in NAFLD remains poorly understood. However, the NHANES database offers valuable data on blood manganese levels and metabolic status for 6278 subjects in the United States, facilitating the study of this relationship.

**Methods:**

To investigate the relationship between blood manganese (Mn) levels and NAFLD, we conducted a *t*-test to compare Mn levels between participants with and without NAFLD. Participants were categorized into quartiles based on their blood Mn levels. We then employed multiple logistic regression analysis and sensitivity analyses to further examine the Mn-NAFLD relationship.

**Results:**

The NAFLD group had a significantly higher blood manganese level (10.0 ± 3.7 μg/L, P < 0.05) than the control group. Stratifying 6278 subjects by blood manganese quartiles showed increased NAFLD odds in higher quartiles (Q2-Q4) vs. Q1 (ORs: 1.49, 1.37, 1.49). The Mn-NAFLD relationship followed an inverted L-shaped curve, peaking at 8.52 μg/L.

**Conclusions:**

Elevated levels of manganese in the blood have been shown to be associated with an increase in the risk of NAFLD, and blood manganese values can be utilized as a marker for assessing NAFLD.

## 1 Introduction

Non-alcoholic fatty liver disease (NAFLD) is characterised by a build-up of liver fat (≥5 %) in the absence of significant alcohol intake or other known causes of liver fat [1]. Driven by shifting dietary patterns, sedentary lifestyles, and metabolic disorders, NAFLD has emerged as a global epidemic affecting 25–30 % of the general population, with substantial socioeconomic burdens [2]. Although many with liver fat have good outlooks [3], up to 20 % progress to non-alcoholic steatohepatitis (NASH), fibrosis, and hepatocellular carcinoma [4]. Presently, few effective NAFLD treatments exist.Current research suggests that NAFLD is closely related to metabolism, but the exact mechanism of influence is unclear,yet the role of trace elements—particularly manganese (Mn)—in modulating disease risk remains underexplored.

Manganese, an essential micronutrient, serves as a cofactor for enzymes involved in carbohydrate/lipid metabolism, antioxidant defense, and mitochondrial function [5]. Both deficiency and excess of Mn have been linked to metabolic disturbances [6]. Experimental studies suggest Mn overload may induce oxidative stress and hepatic lipid deposition, while epidemiological data associate elevated blood Mn with obesity and insulin resistance [7]—key risk factors for NAFLD.

Previous studies on manganese (Mn) and NAFLD, mostly limited to small studies or animal models, report conflicting results on protective versus harmful effects [8,9]. Traditional NAFLD diagnostics (e.g., liver enzymes, ultrasound) lack sensitivity for early steatosis (∼60–70 %), obscuring exposure-disease links [10,11], while dose-response relationships and Mn thresholds remain unclear. To address these knowledge gaps, we leveraged NHANES 2017–2020 data with two key innovations: (1) This study adopts Vibration-Controlled Transient Elastography (VCTE) to measure the Controlled Attenuation Parameter (CAP) (AUROC = 0.96) for precise steatosis quantification [12], overcoming limitations of traditional biomarkers; (2) non-linear dose-response analysis to identify critical Mn thresholds. We hypothesize that blood Mn levels exhibit a biphasic association with NAFLD risk, with a definable threshold beyond which hepatic steatosis risk plateaus.

## 2 Materials and methods

### 2.1 Study population

**Fig. 1:**
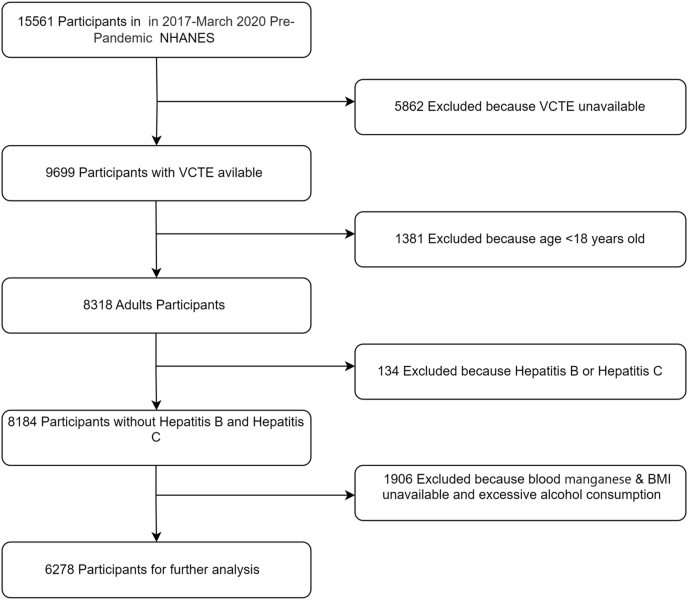
Flow chart of the study.

The National Health And Nutrition Examination Survey (NHANES) database provides information on blood manganese levels and fat metabolism in the U.S. population from January 2017 to March 2020.The National Center for Health Statistics Ethics Review Board approved the NHANES study guidelines and protocols.Informed consent was obtained from participants. Out of 15,561 participants in the NHANES 2017–March 2020 Pre-pandemic cycle, we excluded 5862 for missing VCTE CAP scores, 1381 under 18, 92 with hepatitis C, 42 with hepatitis B, 79 missing BMI data, and 356 missing blood manganese levels. Additionally, 1471 with high alcohol consumption (≥30 g/day for men or ≥20 g/day for women) were excluded, leaving 6278 for analysis (Fig. 1).

### 2.2 Exposures: blood manganese measurements

Blood manganese levels were assessed from whole blood samples collected, frozen at −30 °C, and sent to the CDC in Atlanta, GA for analysis [13,14].

### 2.3 Outcomes: NAFLD

Liver steatosis was measured using FibroScan® at the NHANES Mobile Examination Center [15]. FibroScan uses ultrasound and elastography to assess liver stiffness and the controlled attenuation parameter (CAP) for steatosis.The CAP values range from 100 to 400 dB/m, with higher values indicating increased liver fat [16]. Based on meta-analysis and population studies, Participants with CAP scores ≥274 dB/m were considered to have liver steatosis [17], since patients with excessive alcohol consumption and hepatitis were excluded from our study, our outcome was NAFLD.

### 2.4 Covariates

Demographic information was collected through an interviewer-led questionnaire and included age, gender, race/ethnicity, education, marital status, smoking status, household poverty income ratio (PIR), presence of diabetes, and body mass index (BMI) [18,19].Race/ethnicity was categorized as Non-Hispanic White, Non-Hispanic Black, Mexican American, or other race. Marital status was categorized as married/with a partner or alone. Education level was categorized based on the number of years of education received: less than 9 years, 9–12 years, or more than 12 years. We categorized participants based on their BMI into non-obese (BMI <30.0 kg/m^2^) and obese (BMI*≥*30.0 kg/m^2^). Diabetes status was determined by participants' self-reported history of the condition. Smoking status was divided into three categories: never-smokers (those who have smoked fewer than 100 cigarettes in their lifetime), former-smokers (individuals who have smoked over 100 cigarettes but currently do not smoke), and current smokers (people who have smoked more than 100 cigarettes and continue to smoke).

### 2.5 Statistical analyses

This study analyzed NHANES data from 2017 to 2020. We directly excluded participants with missing values in key variables as shown in Fig. 1.Continuous variables conforming to normal distributions are expressed as mean (standard deviation). Categorical variables are expressed as proportions (%). Analysis of variance (ANOVA) and *t*-test were used to determine whether there was any difference between the NAFLD group and the non-NAFLD group in terms of age, gender, BMI, presence of diabetes, and blood manganese values.

Blood manganese (Mn) concentrations were normally distributed and quartiles were used to describe this variable. In our analysis, we employed multivariable logistic regression models to calculate ORs and 95 % confidence intervals (CIs) for the association between blood manganese levels and NAFLD, accounting for various confounders such as demographic factors and known NAFLD risk factors. The basic model did not adjust for covariates. In Model 1, adjustments were made for age and gender. Model 2 included adjustments for age, gender, race, education attainment, marital status, and family PIR. Model 3 further adjusted for smoking status, BMI and diabetes status on the basis of Model 2. After determining that there was a segmented linear relationship between blood manganese levels and NAFLD, the data were then fitted using a segmented regression model and the inflection point values were determined by the Wald test.

Data processing and analysis were primarily conducted using R software version 3.3.2 and Free Statistics software version 1.9(Beijing Free Clinical Medical Technology Co., LTD. A comprehensive descriptive analysis was performed for all study participants, with two-tailed p-values less than 0.05 considered statistically significant.

## 3 Results

Table 1 presents the baseline characteristics of the 6278 participants in the study. The mean age of the participants was found to be 49.2 years, and 43.5 % of them (2728 participants) had been diagnosed with NAFLD.Individuals in the NAFLD group were found to be on average older, had a higher BMI, a higher prevalence of diabetes, and the blood manganese value of 10.0 ± 3.7 μg/L was elevated in those with NAFLD compared to the non-NAFLD group. This difference was found to be statistically significant.

**Table 1:** Characteristics of participants with or without NAFLD in the NHANES 2017–March 2020 Pre-Pandemic Data.

Characteristic	Overall (n = 6278)	Non-NAFLD (n = 3550)	NAFLD (n = 2728)	*P-value*
**Gender**				<0.001
Male	3010 (47.9)	1577 (44.4)	1433 (52.5)	
Female	3268 (52.1)	1973 (55.6)	1295 (47.5)	
**Age (years)**	49.2 ± 18.3	46.7 ± 19.2	52.4 ± 16.5	<0.001
**Race/ethnicity**				<0.001
Non-Hispanic White	2199 (35.0)	1194 (33.6)	1005 (36.8)	
Non-Hispanic Black	1608 (25.6)	1036 (29.2)	572 (21)	
Mexican American	783 (12.5)	333 (9.4)	450 (16.5)	
Others	1688 (26.9)	987 (27.8)	701 (25.7)	
**Education level (years)**				<0.001
<9	445 (7.5)	214 (6.5)	231 (8.7)	
9–12	2076 (34.9)	1124 (34.2)	952 (35.8)	
>12	3426 (57.6)	1948 (59.3)	1478 (55.5)	
**Marital status**				<0.001
Married or living with a partner	3464 (58.2)	1802 (54.9)	1662 (62.4)	
Living alone	2483 (41.8)	1483 (45.1)	1000 (37.6)	
**Family poverty-income-ratio**				0.051
Low	1598 (29.2)	931 (30.1)	667 (27.9)	
Medium	2133 (39.0)	1161 (37.6)	972 (40.7)	
High	1745 (31.9)	996 (32.3)	749 (31.4)	
**Smoking status**				<0.001
Never	3868 (61.6)	2249 (63.4)	1619 (59.3)	
Former	1412 (22.5)	689 (19.4)	723 (26.5)	
Current	998 (15.9)	612 (17.2)	386 (14.1)	
**Diabetes**				<0.001
No	5331 (84.9)	3241 (91.3)	2090 (76.6)	
Yes	947 (15.1)	309 (8.7)	638 (23.4)	
**BMI (kg/m^2^)**				<0.001
<30.0	3576 (57.0)	2647 (74.6)	929 (34.1)	
*≥*30.0	2702 (43.0)	903 (25.4)	1799 (65.9)	
Blood manganese, ug/L	9.8 ± 3.6	9.7 ± 3.6	10.0 ± 3.7	<0.001

Abbreviations: NAFLD, nonalcoholic fatty liver disease; NHANES, National Health and Nutrition Examination Survey; BMI, body mass index.

In Table 2,when manganese is considered as a continuous variable overall, in the crude model, it exhibits a positive correlation with NAFLD (OR:1.02, 95 % CI: 1.01–1.04, ***P*** = 0.001). After adjusting for potential confounding factors in Models 1, 2, and 3, the correlation between manganese and NAFLD remains stable (***P*** < 0.001).By quartiles of manganese, we observed that in the crude model, compared to Q1, the risk of NAFLD increased in the higher quartiles (Q2, Q3, Q4). After adjusting for potential confounding factors in Models 1, 2, and 3, and in the final Model 3, the risks of NAFLD in Q2, Q3, and Q4 increased by 49 %, 37 %, and 49 % respectively, compared to Q1. The ORs for Q2, Q3, and Q4 were 1.49 (95 % CI: 1.25–1.79), 1.37 (95 % CI: 1.14–1.64), and 1.49 (95 % CI: 1.23–1.8) respectively, with all ***P*** < 0.001.

**Table 2:** Association between blood manganese and NAFLD.

Blood Mn	OR-95 %CI									
	No.of total	No.of event (%)	Crude	*P*-value	Model 1	P-value	Model 2	*P*-value	Model 3	*P*-value
**Overall**	6278	2728 (43.5)	1.02 (1.01–1.04)	0.001	1.05 (1.03–1.07)	<0.001	1.04 (1.02–1.06)	<0.001	1.03 (1.02–1.05)	<0.001
**Quartile**										
**Q1**	1563	592 (37.9)	1(Ref)		1(Ref)		1(Ref)		1(Ref)	
**Q2**	1575	721 (45.8)	1.38 (1.2–1.6)	<0.001	1.56 (1.35–1.8)	<0.001	1.47 (1.25–1.73)	<0.001	1.49 (1.25–1.79)	<0.001
**Q3**	1570	699 (44.5)	1.32 (1.14–1.52)	<0.001	1.54 (1.33–1.78)	<0.001	1.4 (1.19–1.65)	<0.001	1.37 (1.14–1.64)	0.001
**Q4**	1570	716 (45.6)	1.38 (1.19–1.59)	<0.001	1.75 (1.51–2.03)	<0.001	1.49 (1.26–1.77)	<0.001	1.49 (1.23–1.8)	<0.001
P for trend				<0.001		<0.001		<0.001		<0.001

Note:Crude was non-adjusted; Model 1 was adjusted for sociodemographic variables (age and gender); Model 2 was adjusted for Model 1+race/ethnicity,education level, marital status and family PIR; Model 3 was adjusted for Model 2+smoking status, BMI and diabetes.

Abbreviations: CI, confidence interval; OR, odds ratio; Q,quartile; Q1,(1.57–7.40μg/L); Q2(7.41–9.22μg/L),Q3(9.23–11.48μg/L),Q4(11.49–52.07μg/L)Mn,manganese(ug/L).

**Fig. 2:**
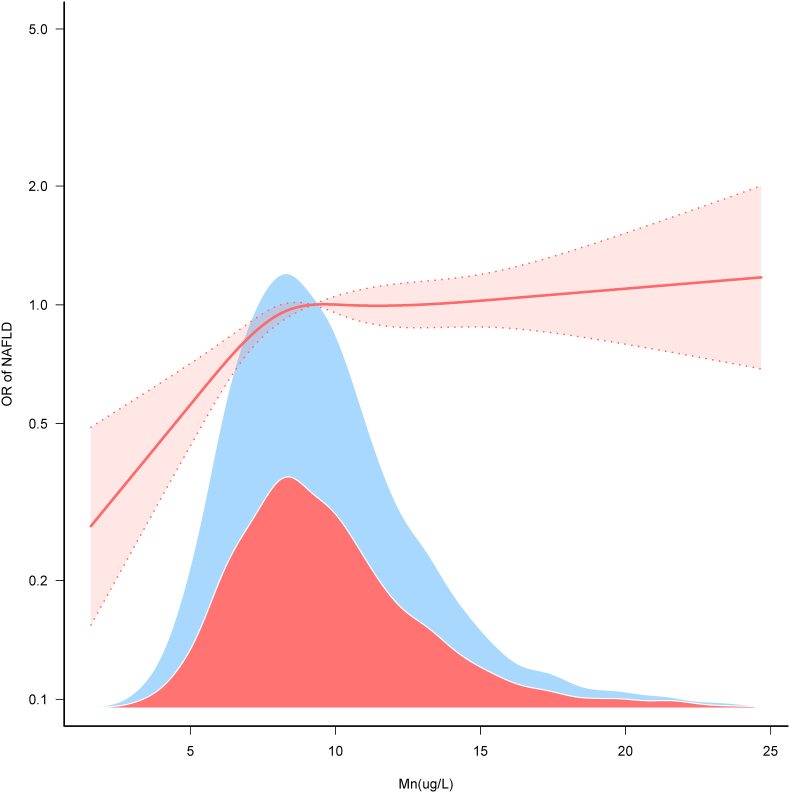
Restricted cubic spline curves for manganese and NAFLD. Note:OR, odds ratio; Mn,manganese. Adjusted Model was adjusted for sociodemographic variables (age and gender),race/ethnicity,education level, marital status and family PIR,smoking status, BMI and diabetes. Only 99.5 % of the date is displayed.

Further analysis using restricted cubic spline plots showed an inverted L-shaped nonlinear relationship (***P*** < 0.001) between blood Mn and NAFLD (Fig. 2), with a significant change in the association at an inflection point of 8.52 μg/L. Below this level, the incidence of NAFLD increased with higher Mn concentrations, while above this point, the association plateaued (Table 3).

**Table 3:** Threshold effect analysis of blood manganese on NAFLD.

Threshold of blood Mn	Adjusted Model	
Mn (ug/L)	OR (95 %CI)	*P*-value
<8.52	*1.202 (1.099–1.315)*	<0.001
*≥8.52*	*1.008 (0.983–1.035)*	0.5251
Likelihood Ratio test	–	<0.001

Note:Abbreviations:CI,confidence interval; OR, odds ratio; Mn,manganese.

Adjusted Model was adjusted for sociodemographic variables (age and gender),race/ethnicity,education level, marital status and family PIR,smoking status, BMI and diabetes. Only 99.5 % of the date is displayed.

**Fig. 3:**
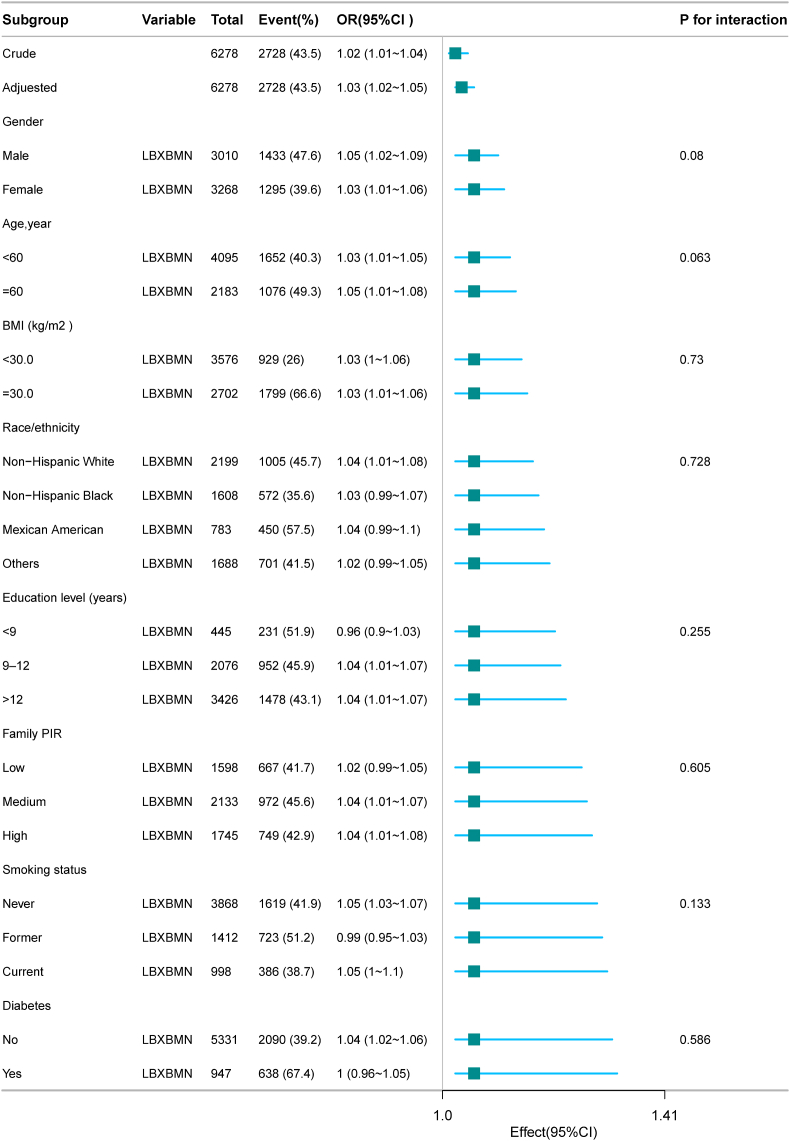
According to fundamental characteristics, the relationship blood manganese and NAFLD. Note:Adjusted Model was adjusted for sociodemographic variables (age and gender),race/ethnicity,education level, marital status and family PIR,smoking status, BMI and diabetes.

Forest plots indicated no significant interactions between blood Mn levels and various subgroups (gender, age, race, education level, family PIR, smoking status, diabetes, BMI) in relation to NAFLD, suggesting a stable positive association across different demographic and risk factor groups (Fig. 3).

## 4 Discussion

Non-alcoholic fatty liver disease (NAFLD) represents an escalating public health concern and currently stands as the most prevalent liver disorder globally [20]. The prevalence of NAFLD, determined using VCTE via controlled attenuation parameters (CAP), was 37.1 % (95 % CI 34.0–40.4), based on 2017–2018 NHANES data [21]. The current cross-sectional study involved 6278 participants from 2017 to 2020 in the U.S., of whom 43.5 % were diagnosed with NAFLD.NAFLD is the accumulation of large amounts of free fatty acids within hepatocytes to form fat droplets that manifest as steatosis. In persistent oxidative stress and inflammation, it leads to persistent liver damage and development of NASH, which progressively worsens and develops into liver fibrosis and cirrhosis in about 40 % of patients with NASH [22]. Therefore it is crucial for early intervention and treatment of NAFLD.

The exact mechanism of NAFLD remains unclear, and is currently found to be closely related to obesity, insulin resistance, and others. The present study in Table 1 similarly shows that BMI and prevalence of diabetes mellitus were higher in the NAFLD group than in the control group (***P*** < 0.05). Unhealthy lifestyle habits, such as diets high in sugar and fat lead to obesity, increased hepatic fat accumulation and hepatic fat de novo lipogenesis (DNL), which directly leads to hepatic steatosis [23]. Normal adipose tissue secretion of lipid transport proteins and leptin is essential for maintaining liver homeostasis, and when hepatic fat accumulation and fibrosis occurs, the secretion of lipid transport proteins decreases, which directly leads to mitochondrial dysfunction further leading to aberrant fat accumulation and accelerating the process of NAFLD. Overexpression of tumor necrosis factor-α (TNF-α) in the livers of obese patients continuously activates IκB kinase β, which mediates the phosphorylation of insulin receptor substrate (IRS) IRS-1 and IRS-2 and disrupts the binding of insulin to its receptor. IR conditions lead to overexpression of SREBP-1 (sterol regulatory element binding protein-1), which triggers upregulation of de novo lipogenesis (DNL), resulting in Impaired inhibition of lipolysis in adipose tissue (AT) and the production of large amounts of free fatty acids, which eventually accumulate in the liver and kidney [24], further aggravating NAFLD.

Among the 6278 participants in the present study, the mean blood manganese level in NAFLD patients was found to be 10.0 ± 3.7 μg/L. This value was significantly higher than that observed in the non-NAFLD group (***P*** < 0.05). Manganese is a crucial yet minor element in the human body, primarily acquired through diet and water consumption. It is absorbed by the gastrointestinal tract and subsequently transported to mitochondria-rich tissues within the body, especially in the liver, manganese is rapidly concentrated. Furthermore, Mn plays a vital role in the synthesis and activation of numerous enzymes, including oxidoreductases, transferases, hydrolases, lyases, isomerases, and ligases [25].

In order to study the effect of manganese on lipid metabolism, Gubert [26] utilized *Caenorhabditis elegans* as a biological model to observe the effects of manganese exposure on lipid storage and metabolic activity in nematodes. This study found that manganese increased the expression of sbp-1 transcription factor and decreased the expression of let-363 protein kinase, thereby affecting lipid metabolism.Yang [27] found that dietary manganese supplementation could promote the breakdown of fat triglycerides by inhibiting the expression of sterol regulatory element-binding protein 1 (SREBP1) and SCD, as well as reducing the activity of stearoyl-CoA desaturase, and by affecting lipase ATGL expression and activity.

Manganese activates enzymes such as pyruvate carboxylase, increases insulin secretion, improves glucose tolerance under dietary stress conditions, reduces oxidative stress (ROS) and NADPH oxidase activity, and decreases the risk of endocrine dysfunction in diabetes [28]. Gamarra demonstrated that manganese modulates hepatic insulin sensitivity and glucose tolerance through a mouse model [29]. Riseberg demonstrated a relationship between manganese and glucose levels by studying data from 1478 cases in the San Luis Valley Diabetes Study (SLVD). The study found that manganese was associated with low fasting blood glucose levels [30].

This shows that Mn feasibly affects the development of NAFLD by influencing lipid metabolism and glucose metabolism. In the present study through multivariate and sensitivity analysis, we found that blood manganese level is positively correlated with NAFLD as shown in Table 2. As shown in the restricted cubic spline plot, this relationship forms an inverted L-shape with a turning point of 8.52 μg/L. Therefore, maintaining blood manganese levels at or below a certain threshold may help reduce the risk of developing NAFLD.

Our study reveals that excessive manganese exposure correlates with increased risk of hepatic steatosis. These findings support targeted public health strategies, including manganese exposure monitoring in high-risk populations (e.g., industrial workers, residents near manganese-polluted areas) and evidence-based dietary guidelines to optimize manganese intake. Clinically, blood manganese quantification could enhance NAFLD risk stratification as an adjunct screening tool, particularly in individuals with metabolic comorbidities. Future research should prioritize elucidating manganese's dual roles in lipid metabolism and exploring therapeutic interventions modulating manganese homeostasis in order to prevent and reduce the incidence of NAFLD.

However, this study has some limitations that need to be acknowledged. Despite considering numerous variables in the model, potential unknown confounders may still exist due to gaps or missing data in the NHANES database. The cross-sectional nature of the study further limits our ability to draw causal conclusions. Moreover, since the data are sourced exclusively from the NHANES database and represent only the U.S. population, caution is advised when generalizing these results to other international populations.

## 5 Conclusion

This study revealed a positive association between blood manganese levels and NAFLD diagnosed by VCTE, and the risk of NAFLD incidence gradually leveled off when manganese levels were greater than 8.52μg/L. Our results suggest that blood manganese quantification is a promising auxiliary biomarker for risk stratification of NAFLD. Maintaining manganese intake below the established threshold (8.52 μg/L) may be a preventive strategy, especially in high-risk individuals with metabolic comorbidities.

## CRediT authorship contribution statement

**Qian Xue:** Writing – review & editing, Writing – original draft, Visualization, Validation, Supervision, Software, Resources, Project administration, Methodology, Investigation, Funding acquisition, Formal analysis, Data curation, Conceptualization. **Hongju Chen:** Writing – review & editing, Software, Resources, Project administration, Funding acquisition, Formal analysis, Data curation, Conceptualization.

## Date availability statement

Public datasets used in this study are available online. The repository names and accession numbers are available at http://www.cdc.gov/nchs/nhanes.htm (accessed: Feb. 04, 2024).

## Fund information

This research did not receive any specific grant from funding agencies in public, commercial,or not-for-profit sectors.

## Conflicts of interest

The authors declare no conflict of interest.

## References

[1] Vallianou N., Liu J., Dalamaga M. (2019). What are the key points in the association between the gut microbiome and nonalcoholic fatty liver disease?. Metab Open.

[2] Huang D.Q., El-Serag H.B., Loomba R. (2021). Global epidemiology of NAFLD-related HCC: trends, predictions, risk factors and prevention. Nat Rev Gastroenterol Hepatol.

[3] Dam-Larsen S. (2004). Long term prognosis of fatty liver: risk of chronic liver disease and death. Gut.

[4] Friedman S.L., Neuschwander-Tetri B.A., Rinella M., Sanyal A.J. (2018). Mechanisms of NAFLD development and therapeutic strategies. Nat Med.

[5] Maret W. (2016). The metals in the biological periodic system of the elements: concepts and conjectures. Int J Mol Sci.

[6] Li L., Yang X. (2018). The essential element manganese, oxidative stress, and metabolic diseases: links and interactions. Oxid Med Cell Longev.

[7] Liu J., Tan L., Liu Z., Shi R. (2022). Blood and urine manganese exposure in non-alcoholic fatty liver disease and advanced liver fibrosis: an observational study. Environ Sci Pollut Res.

[8] Wu L., Lan Y., Yu Z. (2023). Blood manganese and non-alcoholic fatty liver disease in a high manganese exposure area in China. J Health Popul Nutr.

[9] Rezazadeh Alireza, Yazdanparast R. (1996). Prevention of nonalcoholic steatohepatitis in rats by two manganese-salen complexes. Iran Biomed J.

[10] Schwenzer N.F., Springer F., Schraml C., Stefan N., Machann J., Schick F. (2009). Non-invasive assessment and quantification of liver steatosis by ultrasound, computed tomography and magnetic resonance. J Hepatol.

[11] Torres D.M., Harrison S.A. (2008). Diagnosis and therapy of nonalcoholic steatohepatitis. Gastroenterology.

[12] EASL–EASD–EASO (2016). Clinical Practice Guidelines for the management of non-alcoholic fatty liver disease. J Hepatol.

[13] Eddowes P.J., Sasso M., Allison M. (2019). Accuracy of FibroScan controlled attenuation parameter and liver stiffness measurement in assessing steatosis and fibrosis in patients with nonalcoholic fatty liver disease. Gastroenterology.

[14] Gibson R.S., Anderson V.P. (2009). A review of interventions based on dietary diversification or modification strategies with the potential to enhance intakes of total and absorbable zinc. Food Nutr Bull.

[15] Hwang J.J., Park M.H., Choi S.Y., Koh J.Y. (2005). Activation of the trk signaling pathway by extracellular zinc. J Biol Chem.

[16] Eddowes P.J., Sasso M., Allison M. (2019). Accuracy of FibroScan controlled attenuation parameter and liver stiffness measurement in assessing steatosis and fibrosis in patients with nonalcoholic fatty liver disease. Gastroenterology.

[17] Siddiqui M.S., Vuppalanchi R., Van Natta M.L. (2019). Vibration-controlled transient elastography to assess fibrosis and steatosis in patients with nonalcoholic fatty liver disease. Clin Gastroenterol Hepatol.

[18] Vegel A.J., Benden D.M., Borgert A.J., Kallies K.J., Kothari S.N. (2017). Impact of obesity on cesarean delivery outcomes. Wis Med J: off publ State Med Soc Wis.

[19] Ghoneim S., Butt M.U., Hamid O., Shah A., Asaad I. (2020). The incidence of COVID-19 in patients with metabolic syndrome and non-alcoholic steatohepatitis: a population-based study. Metab Open.

[20] Barbara M., Mindikoglu A.L. (2021). The role of zinc in the prevention and treatment of nonalcoholic fatty liver disease. Metabol Open.

[21] Ciardullo S., Perseghin G. (2021). Prevalence of NAFLD, MAFLD and associated advanced fibrosis in the contemporary United States population. Liver Int.

[22] Younossi Z.M., Koenig A.B., Abdelatif D., Fazel Y., Henry L., Wymer M. (2016). Global epidemiology of nonalcoholic fatty liver disease—meta‐analytic assessment of prevalence, incidence, and outcomes. Hepatology.

[23] Pal S.C., Méndez-Sánchez N. (2023). Insulin resistance and adipose tissue interactions as the cornerstone of metabolic (dysfunction)-associated fatty liver disease pathogenesis. World J Gastroenterol.

[24] Yang K., Cui X., Hu Y. (2024). Dietary manganese supplementation decreases hepatic lipid deposition by regulating gene expression and enzyme activity involved in lipid metabolism in the liver of broilers. J Anim Sci.

[25] Gubert P., Puntel B., Lehmen T. (2018). Metabolic effects of manganese in the nematode Caenorhabditis elegans through DAergic pathway and transcription factors activation. Neurotoxicology.

[26] Gubert P., Puntel B., Lehmen T. (2018). Metabolic effects of manganese in the nematode Caenorhabditis elegans through DAergic pathway and transcription factors activation. Neurotoxicology.

[27] Yang K., Cui X., Hu Y. (2024). Dietary manganese supplementation decreases hepatic lipid deposition by regulating gene expression and enzyme activity involved in lipid metabolism in the liver of broilers. J Anim Sci.

[28] Burlet E., Jain S.K. (2017). Manganese supplementation increases adiponectin and lowers ICAM-1 and creatinine blood levels in Zucker type 2 diabetic rats, and downregulates ICAM-1 by upregulating adiponectin multimerization protein (DsbA-L) in endothelial cells. Mol Cell Biochem.

[29] Gamarra J., Xie Y., Higuchi S., Haeusler R. (2024). 280-OR: manganese regulates hepatic insulin sensitivity and glucose tolerance. Diabetes.

[30] Riseberg E., Chui K., James K.A., Melamed R., Alderete T.L., Corlin L. (2022). A longitudinal study of exposure to manganese and incidence of metabolic syndrome. Nutrients.

